# Metabolic Advantage of 25(OH)D_3_ versus 1,25(OH)_2_D_3_ Supplementation in Infantile Nephropathic Cystinosis-Associated Adipose Tissue Browning and Muscle Wasting

**DOI:** 10.3390/cells11203264

**Published:** 2022-10-17

**Authors:** Ping Zhou, Wai W. Cheung, Alex Gonzalez, Venya Vaddi, Eduardo A. Oliveira, Robert H. Mak

**Affiliations:** 1 Division of Pediatric Nephrology, Rady Children’s Hospital, University of California, San Diego, CA 92093, USA; 2 Department of Pediatric Nephrology and Rheumatology, Sichuan Provincial Maternity and Child Health Care Hospital and The Affiliated Women’s and Children’s Hospital of Chengdu Medical College, Chengdu 610031, China; 3 Integrative Biology & Physiology, University of California, Los Angeles, CA 90095, USA; 4 Department of Pediatrics, Health Sciences Postgraduate Program, School of Medicine, Federal University of Minas Gerais (UFMG), Belo Horizonte 30310-100, MG, Brazil

**Keywords:** infantile nephropathic cystinosis, cachexia, adipose tissue browning, muscle wasting, vitamin D insufficiency, 25(OH)D_3_, 1,25(OH)_2_D_3_

## Abstract

Manifestations of infantile nephropathic cystinosis (INC) often include cachexia and deficiency of circulating vitamin D metabolites. We examined the impact of 25(OH)D_3_ versus 1,25(OH)_2_D_3_ repletion in *Ctns* null mice, a mouse model of INC. Six weeks of intraperitoneal administration of 25(OH)D_3_ (75 μg/kg/day) or 1,25(OH)_2_D_3_ (60 ng/kg/day) resulted in *Ctns^−/−^* mice corrected low circulating 25(OH)D_3_ or 1,25(OH)_2_D_3_ concentrations. While 25(OH)D_3_ administration in *Ctns^−/−^* mice normalized several metabolic parameters characteristic of cachexia as well as muscle function in vivo, 1,25(OH)_2_D_3_ did not. Administration of 25(OH)D_3_ in *Ctns^−/−^* mice increased muscle fiber size and decreased fat infiltration of skeletal muscle, which was accompanied by a reduction of abnormal muscle signaling pathways. 1,25(OH)_2_D_3_ administration was not as effective. In conclusion, 25(OH)D_3_ supplementation exerts metabolic advantages over 1,25(OH)_2_D_3_ supplementation by amelioration of muscle atrophy and fat browning in *Ctns^−/−^* mice.

## 1. Introduction

Infantile nephropathic cystinosis (INC), a genetic chronic kidney disease (CKD), results from cystinosin (CTNS) mutations and involves the deposition of cystine crystals in multiple organs [1,2]. Children with INC present with myopathy and neuromuscular abnormalities such as swallowing difficulty. Currently, there are no known treatments to address these comorbidities [3,4]. We described the cachexic phenotype in *Ctns* null mice, an animal model of INC, with extensive fat browning and muscle atrophy [5]. White fat stores energy, whereas brown fat utilizes stored energy during thermogenesis to produce heat [6]. White fat browning (a process in which white adipocytes phenotypically change to brown-fat-like cells) has been implicated in the progression of cachexia, as demonstrated by recent studies [7,8,9,10]. The metabolism of skeletal muscle and brown fat are connected as brown fat modulates the function of skeletal muscle through the release of myostatin, a powerful inhibitor of muscle function [11]. Importantly, fat browning precedes muscle wasting in cancer and CKD [12,13]. Characterizing the complex interactions between various energy-wasting pathways involved in cachexia represents a key step towards establishing effective clinical therapies for this profound complication in patients with INC.

Vitamin D acts as an anti-proliferative factor in various tissues (such as fat and muscle) and physiological systems (such as renal, cardiovascular, and immune systems) [14]. Insufficiency of vitamin D is present in numerous pathological conditions [15]. INC patients commonly present with insufficiency of 25(OH)D_3_ and 1,25(OH)_2_D_3_ [16,17,18]. Previously, we found that the administration of 25(OH)D_3_ and 1,25(OH)_2_D_3_ reduced the effect of cachexia and white adipose tissue (WAT) browning in *Ctns^−/−^* mice [19]. 1α-hydroxylase, which is present in the kidney as well as locally in muscle, activates the metabolite 25(OH)D_3_ (the most prevalent metabolite in circulation) to circulating 1,25(OH)_2_D_3_, which binds the vitamin D receptor (VDR) to exert the downstream responses [20,21,22]. Interestingly, 25(OH)D_3_ shows strong in vivo and ex vivo effects by itself [23,24,25,26,27]. Here, we compared 25(OH)D_3_ versus 1,25(OH)_2_D_3_ administration in *Ctns^−/−^* mice, specifically focusing on fat and muscle abnormalities.

## 2. Materials and Methods

### 2.1. Study Design

Twelve-month-old male, c57BL/6 wild-type (WT) mice and *Ctns^−/−^* mice (c57BL/6 genetic background) [28] were subcutaneously supplemented with 25(OH)D_3_ (Sigma, Northbrook, IL, USA, Catalog 739,650-1ML, 25, 50 or 75 μg/kg/day), 1,25(OH)_2_D_3_ (Sigma, Northbrook, IL, USA, Catalog 740,578-1ML, 20, 40 or 60 ng/kg/day) or vehicle (ethylene glycol) for six weeks by using Alzet mini-osmotic pump model 2006 (Durect Corporation, Cupertino, CA, USA). We used both *ad libitum* and pair-feeding strategy. Mice were fed with rodent diet 5015 (catalog 0001328, LabDiet, St Louis, MO, USA). This study was approved and performed at University of California, San Diego.

### 2.2. Measuremnt of Lean and Fat Mass

Body composition was determined by using EchoMRI-100™ (Echo Medical System, Huston, TX, USA) [5,19].

### 2.3. Resting Metabolic Rate

This was assessed by using Oxymax calorimetry (Columbus Instruments, Columbus, OH, USA) during the daytime (0900-1700) [5,19].

### 2.4. Mouse Muscle Function

Rotarod activity (model RRF/SP, Accuscan Instrument, Columbus, OH, USA) and forelimb grip strength (Model 47106, UGO Basile, Gemonio, VA, Italy) in mice [5,19] were assessed.

### 2.5. Serum and Blood Chemistry

At sacrifice, BUN, electrolytes, 25(OH)D_3_, and 1,25(OH)_2_D_3_ were measured (Supplemental Table S1). Serum creatinine was measured as previously reported [28].

### 2.6. Protein Assay for Muscle and Adipose Tissue

Protein concentration of the tissue homogenate was analyzed using a Pierce BAC Protein Assay Kit (catalog 23227, Thermo Scientific, Waltham, MA, USA).

### 2.7. Fiber Size and Fatty Infiltration of Gastrocnemius

We used Image J software (https://imagej.nih.gob/ij/download.html) (accessed on 13 January 2021) to determine gastrocnemius muscle fiber size [5,19]. In addition, Oil Red O incubation was used to quantify fatty infiltration in skeletal muscle using ImageJ software [29,30].

### 2.8. Muscle Cystine Content Measurement

Muscle cystine contents of gastrocnemius was measured according to published protocols [31,32] by mass spectrometry.

### 2.9. Muscle RNAseq Analysis

RNAseq analysis previously identified differentially expressed muscle genes in *Ctns^−/−^* mice relative to WT mice [19]. In this study, we performed qPCR analysis for these muscle genes in the different experimental groups.

### 2.10. Quantitative Real-Time PCR

We reverse transcribed 3 µg of total RNA to cDNA. Quantitative real-time RT-PCR of target genes was performed as previously published [5,19]. Information for primers are provided (Supplemental Table S2).

### 2.11. Statistics

Statistics analysis was performed using GraphPad Prism version 9.3.1 (GraphPad Software, San Diego, CA, USA). Post hoc analysis was performed with Tukey’s test.

## 3. Results

### 3.1. Supplementation of 25(OH)D_3_ or 1,25(OH)_2_D_3_ Replenishes Serum 25(OH)D_3_ or 1,25(OH)_2_D_3_ Concentration in Ctns^−/−^ Mice

Twelve-month-old *Ctns^−/−^* mice showed significantly lower serum concentration of both 25(OH)D_3_ and 1,25(OH)_2_D_3_. We determined the optimal doses of 25(OH)D_3_ and 1,25(OH)_2_D_3_ needed to normalize serum concentrations of these molecules in *Ctns^−/−^* mice, (Supplemental Tables S3–S5). We observed that supplementation of 25(OH)D_3_ (75 μg/kg/day for 6 weeks) normalized serum concentration of 25(OH)D_3_ as well as significantly increased but not normalized serum concentration of 1,25(OH)_2_D_3_ in *Ctns^−/−^* mice whereas supplementation of 1,25(OH)_2_D_3_ (60 ng/kg/day for 6 weeks) normalized serum concentration of 1,25(OH)_2_D_3_ but did not increase serum concentration of 25(OH)D_3_ in *Ctns^−/−^* mice.

### 3.2. Repletion of 25-Hydroxyvitamin D_3_ Normalizes Caloric Intake and Weight Gain in Ctns^−/−^ Mice

**Figure 1:**
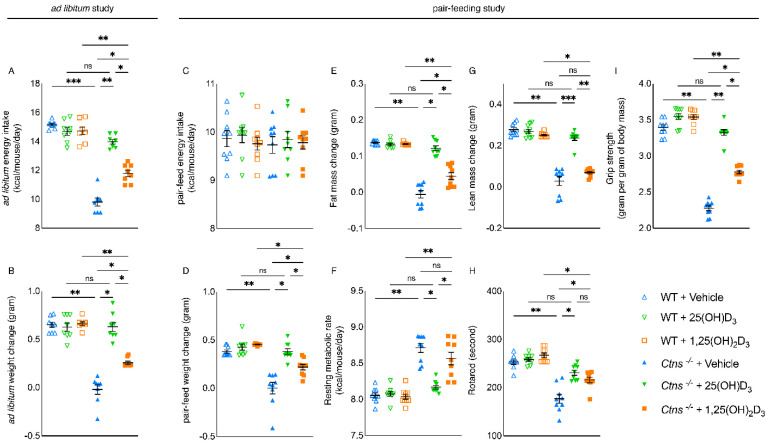
Repletion of 25(OH)D_3_ corrects cachexia in *Ctns^−/−^* mice. We have performed two studies. For the first study, *Ctns^−/−^* and WT mice were given 25(OH)D_3_ (75 µg/kg/day), 1,25(OH)_2_D_3_ (60 ng/kg/day), or vehicle (ethylene glycol), respectively, for six weeks. All mice were fed *ad libitum*. We calculated *ad libitum* caloric intake (**A**) and recorded weight change in mice (**B**). For the second experiment, we employed a diet restrictive strategy. *Ctns^−/−^* + Vehicle mice were given an *ad libitum* amount of food whereas other groups of mice were given an equivalent amount of food (**C**). Weight gain, fat and lean content, resting metabolic rate, and in vivo muscle function (rotarod and grip strength) were measured in mice (**D**–**I**). Data are expressed as mean ± SEM. Results of *Ctns^−/−^* + Vehicle, *Ctns^−/−^* + 25(OH)D_3_, and *Ctns^−/−^* + 1,25(OH)_2_D_3_ mice were compared to those of WT + Vehicle, WT + 25(OH)D_3_, and WT + 1,25(OH)_2_D_3_ mice, respectively. In addition, results of *Ctns^−/−^* + Vehicle were compared to those of *Ctns^−/−^* + 25(OH)D_3_ and *Ctns^−/−^* + 1,25(OH)_2_D_3_ mice, respectively. Furthermore, results of *Ctns^−/−^* + 25(OH)D_3_ mice were compared to those of *Ctns^−/−^* + 1,25(OH)_2_D_3_ mice. Specific *p*-values are shown above the bar. ns signifies not significant, * *p* < 0.05, ** *p* < 0.01, *** *p* < 0.001.

In the first series of experiments, all mice were fed *ad libitum*. Serum chemistry of the mice is listed in Table 1. While supplementing 1,25(OH)_2_D_3_ did not have an effect, repletion of 25(OH)D_3_ in *Ctns^−/−^* mice corrected anorexia (Figure 1A) and normalized weight gain (Figure 1B).

### 3.3. Repletion of 25-Hydroxyvitamin D_3_ Improves Energy Homeostasis in Ctns^−/−^ Mice

In the second series of experiments, we utilized a food restrictive strategy to study the effects of restoring 25(OH)D_3_ versus 1,25(OH)_2_D_3_ levels in *Ctns^−/−^* mice without the effects of different nutritional intake. *Ctns^−/−^* + Vehicle mice were fed *ad libitum* and we determined their daily *ad libitum* caloric intake. The other mouse groups received an energy intake amount equal to that of *Ctns^−/−^* + Vehicle (Figure 1C). Serum chemistry of the mice is listed in Table 2. Replenishing serum 25(OH)D_3_ concentration normalized weight gain, fat mass content, resting metabolic rate, lean mass content, and muscle function (shown by rotarod and grip strength) in *Ctns^−/−^* mice; whereas replenishing serum 1,25(OH)_2_D_3_ concentration improved but not normalize these parameters in *Ctns^−/−^* mice (Figure 1D–I).

### 3.4. Repletion of 25-Hydroxyvitamin D_3_ Improves Adipose Tissue and Skeletal Muscle Energy Homeostasis in Ctns^−/−^ Mice

**Figure 2:**
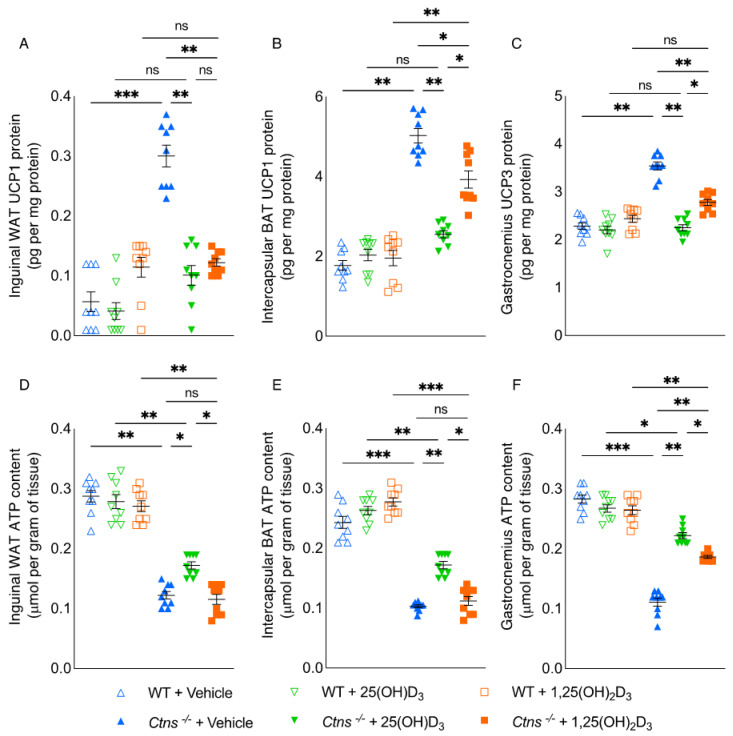
Energy homeostasis improved in skeletal muscle and adipose tissue following repletion of 25-hydroxyvitamin D_3_ in *Ctns^−/−^* mice. UCP content (**A**–**C**) and ATP content (**D**–**F**) in various tissues were measured. Results are expressed and analyzed as in Figure 1. ns signifies not significant, * *p* < 0.05, ** *p* < 0.01, *** *p* < 0.001.

We analyzed the effects of vitamin D repletion in *Ctns^−/−^* mice on energy homeostasis in adipose tissue and skeletal muscle. In WAT, BAT, and the gastrocnemius of *Ctns^−/−^* mice, the protein content of UCPs was significantly higher whereas ATP content was significantly lower relative to WT control mice (Figure 2). The protein content of UCPs was normalized in WAT, BAT, and the gastrocnemius with the repletion of 25(OH)D_3_ in *Ctns^−/−^* mice (Figure 2A–C). Additionally, the improvement in ATP content in WAT, BAT, and the gastrocnemius was significantly better with the repletion of 25(OH)D_3_ compared to the repletion of 1,25(OH)_2_D_3_ in *Ctns^−/−^* mice (Figure 2D–F).

### 3.5. Repletion of 25-Hydroxyvitamin D_3_ Mitigates White Adipose Tissue Browning in Ctns^−/−^ Mice

**Figure 3:**
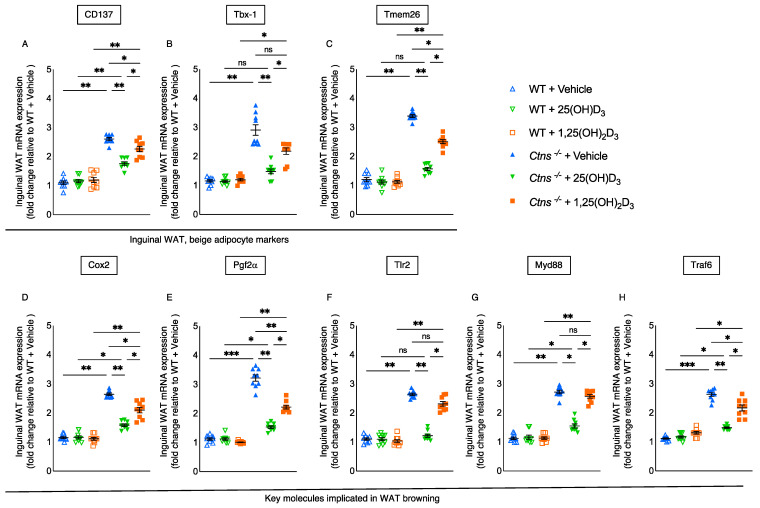
White adipose tissue browning in *Ctns^−/−^* mice was reduced with the repletion of 25-hydroxyvitamin D_3_. qPCR was used to measure gene expression levels in inguinal WAT, specifically for beige adipocyte markers (CD137, Tbx−1 and Tmem26) (**A**–**C**, respectively) and important molecules that mediate WAT browning (Cox2, Pgf2α, Tlr2, Myd88 and Traf6) (**D**–**H**, respectively). Final results were expressed in arbitrary units, with one unit being the mean level in WT + Vehicle mice. Results are expressed and analyzed as in Figure 1. ns signifies not significant, * *p* < 0.05, ** *p* < 0.01, *** *p* < 0.001.

Beige adipocyte cell surface markers (CD137, Tbx1, and Tmem26) expression in inguinal WAT was significantly more reduced with the repletion of 25(OH)D_3_ levels than with the repletion of 25(OH)D_3_ *Ctns*^−/−^ mice (Figure 3A–C). In WAT, *de novo* browning recruitment is promoted by the activation of Cox2/Pgf2α pathway and Toll-like receptor Tlr2 and adaptor molecules, such as Myd88 and Traf6 [33]. The expression of inguinal WAT Cox2, Pgf2α, Tlr2, Myd88, and Traf6 was significantly more reduced with the repletion of 25(OH)D_3_ than with the repletion of 1,25(OH)_2_D_3_ in *Ctns^−/−^* mice (Figure 3D–H).

### 3.6. Repletion of 25-Hydroxyvitamin D_3_ Decreases WAT Thermogenic Gene Expression in Ctns^−/−^ Mice

**Figure 4:**
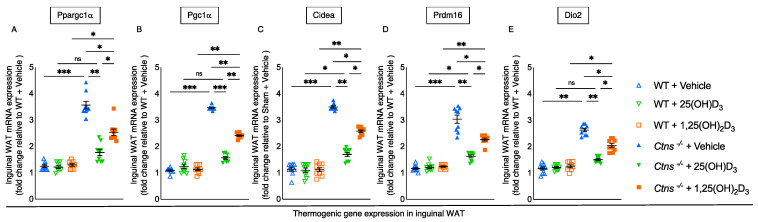
White adipose tissue thermogenic gene expression was normalized or decreased with the repletion of 25-hydroxyvitamin D_3_ in *Ctns^−/−^* mice. In inguinal WAT, qPCR was used to measure thermogenic gene (Ppargc1α, Pgc1α, Cidea, Prdm16 and Dio2) expression (**A**–**E**, respectively). Results are expressed and analyzed as in Figure 3. ns signifies not significant, * *p* < 0.05, ** *p* < 0.01, *** *p* < 0.001.

Compared to WT mice, there was significantly increased expression of thermogenesis genes (Ppargc1α, Pgc1α, Cidea, Prdm16, and Dio2) in inguinal WAT of *Ctns^−/−^* mice. The expression of inguinal WAT genes was normalized (Ppargc1α, Pgc1α, and Dio2) or decreased (Cidea and Prdm16) with the repletion of 25(OH)D_3._ With repletion of 1,25(OH)_2_D_3_ in *Ctns^−/−^* mice, there was improvement but not normalization of these genes. (Figure 4).

### 3.7. Repletion of 25-Hydroxyvitamin D_3_ Ameliorates Muscle Wasting Signaling Pathways in Ctns^−/−^ Mice

**Figure 5:**
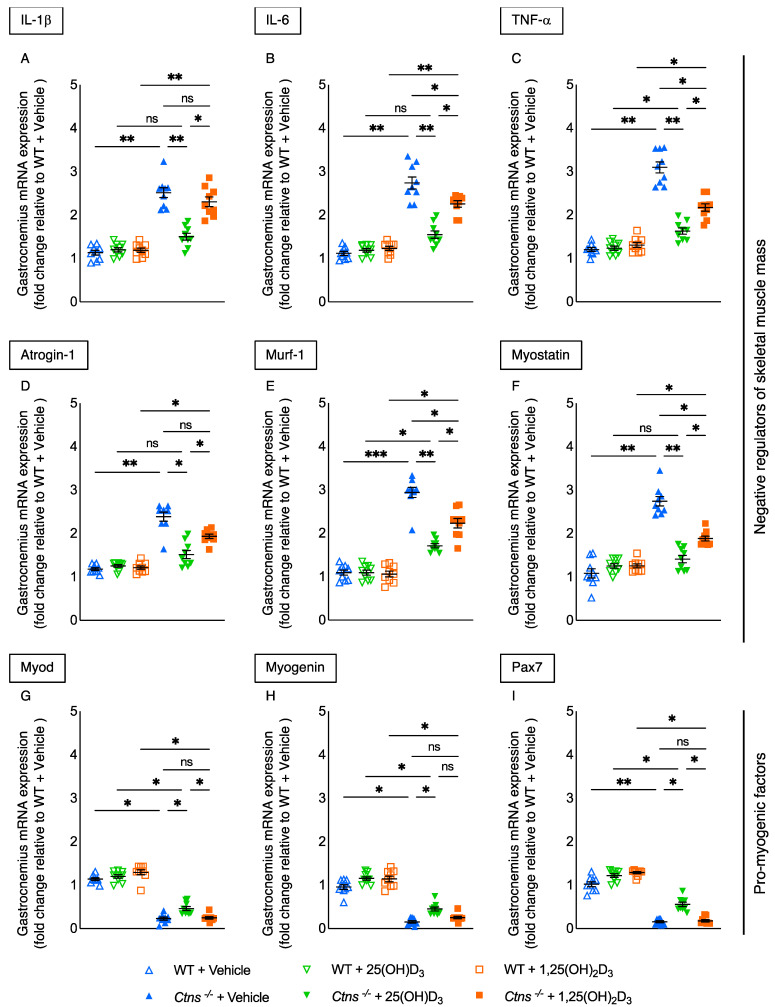
Signaling pathway abnormalities implicated in muscle wasting were improved or normalized with repletion of 25-hydroxyvitamin D_3_ in *Ctns^−/−^* mice. qPCR was used to determine expression levels of negative regulators of skeletal muscle mass (IL-1β, IL-6, TNF-α, Atrogin-1, Murf-1, and Myostatin) and pro-myogenic factors (Myod, Myogenin, and Pax7) in gastrocnemius muscle (**A**–**I**, respectively). Results are expressed and analyzed as in Figure 3. ns signifies not significant, * *p* < 0.05, ** *p* < 0.01, *** *p* < 0.001.

Gastrocnemius expression of inflammatory cytokine was normalized (IL-1β and IL-6) or significantly decreased (TNF-α) with the repletion of 25(OH)D_3_ in *Ctns^−/−^* mice (Figure 5A–C). Additionally, the expression of negative regulators of skeletal muscle mass (Atrogin-1, Murf-1, and Myostatin) in the gastrocnemius was normalized or decreased by the repletion of 25(OH)D_3_ in *Ctns^−/−^* mice (Figure 5D–F), which was a significantly stronger effect than observed with the repletion of 1,25(OH)_2_D_3_. Furthermore, there was increased expression of pro-myogenic factors (Myod, Myogenin and Pax7) with the repletion of 25(OH)D_3_ (Figure 5G–I). There were no significant changes in expression of these genes with the repletion 1,25(OH)_2_D_3_.

### 3.8. Repletion of 25-Hydroxyvitamin D_3_ Increases Muscle Fiber Size in Ctns^−/−^ Mice

**Figure 6:**
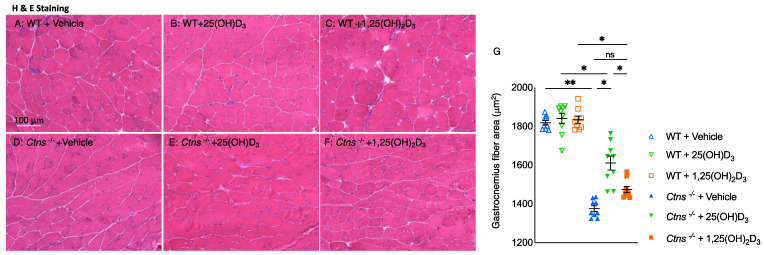
Gastrocnemius fiber size significantly increased with repletion of 25-hydroxyvitamin D_3_ in *Ctns^−/−^* mice. Representative photomicrographs of the gastrocnemius with H&E staining (**A**–**F**). Average gastrocnemius cross-sectional area was measured (**G**). Results are expressed and analyzed as in Figure 1. ns signifies not significant, * *p* < 0.05, ** *p* < 0.01.

While investigating the effect of vitamin D repletion on skeletal muscle morphology in *Ctns^−/−^* mice, we found that the average cross-sectional area of the gastrocnemius increased significantly when restoring the levels of 25(OH)D_3_ but not 1,25(OH)_2_D_3_ (Figure 6).

### 3.9. Repletion of 25-Hydroxyvitamin D_3_ Decreases Muscle Fat Infiltration in Ctns^−/−^ Mice

**Figure 7:**
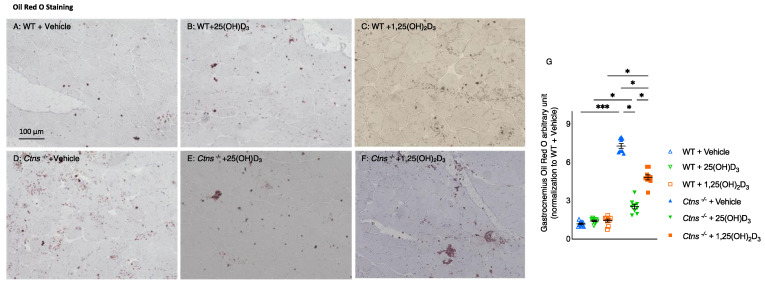
Fatty infiltration in skeletal muscle was reduced with repletion of 25-hydroxyvitamin D_3_ in *Ctns^−/−^* mice. Visualization of the quantification of fatty infiltration by Oil Red O analysis in the gastrocnemius muscle (**A**–**F**). Final results were expressed in arbitrary units, with one unit being the mean staining intensity in WT + Vehicle mice (**G**). Results are expressed and analyzed as in Figure 1. * *p* < 0.05, *** *p* < 0.001.

Fatty infiltration in skeletal muscle was significantly decreased with the repletion of 25(OH)D_3_ compared to repletion of 1,25(OH)_2_D_3_ (Figure 7).

### 3.10. Muscle Content of Cystine in Ctns^−/−^ Mice

**Figure 8:**
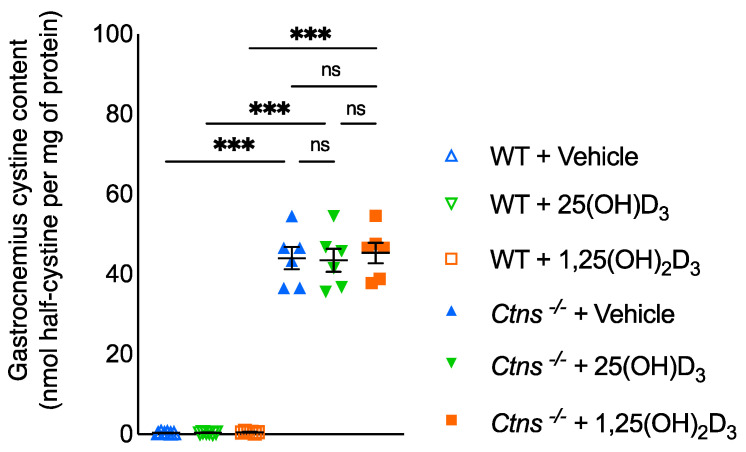
Gastrocnemius cystine content in the mice. Results are expressed and analyzed as in Figure 1. ns signifies not significant, *** *p* < 0.001.

We measured gastrocnemius cystine in the experimental mice. Muscle cystine content was significantly increased in *Ctns^−/−^* mice (Figure 8). Supplementation of 25(OH)D_3_ or 1,25(OH)_2_D_3_ did not influence muscle cystine content in *Ctns^−/−^* mice.

### 3.11. Molecular Mechanism of 25-Hydroxyvitamin D_3_ Repletion by RNAseq Analysis

**Figure 9:**
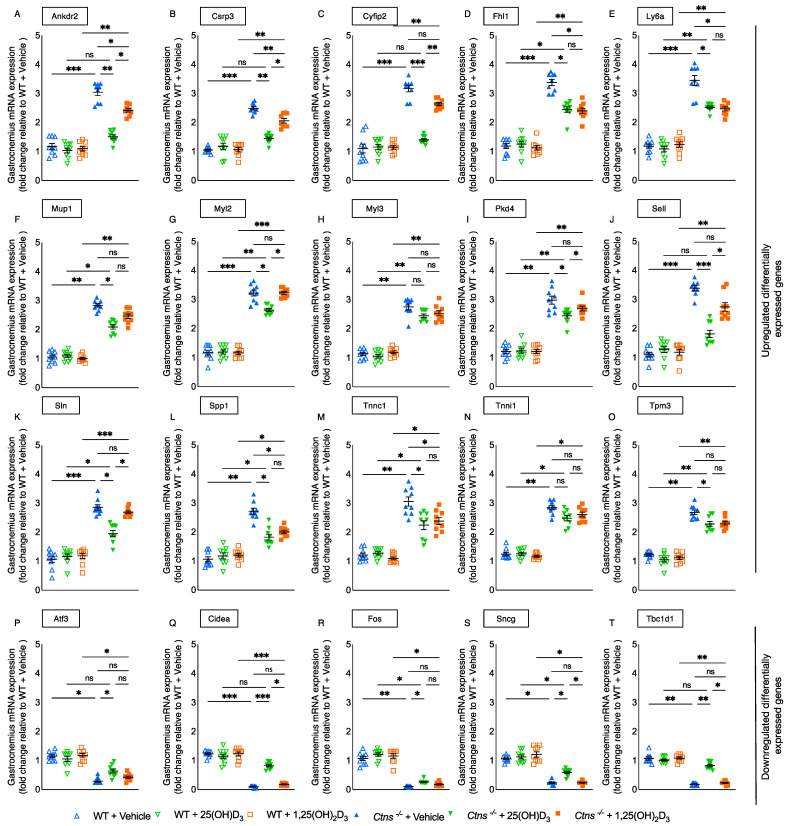
Gastrocnemius muscle gene expression was decreased with repletion of 25-hydroxyvitamin D_3_ in *Ctns^−/−^* mice. Repletion of 25-hydroxyvitamin D_3_ significantly decreased or normalized (Ankdr2, Csrp3, Cyfip2, Fhl1, Ly6a, Mup1, Myl2, Pkd4, Sell, Sln, Spp1, Tnnc1, and Tpm3) (**A**–**O**, respectively) as well as (Atf3, Cidea, Fos, Sncg, and Tbc1d1) (**P**–**T**, respectively) muscle gene expression, but repletion of 1,25(OH)_2_D_3_ did not, in *Ctns^−/−^* mice. Nonsignificant changes were observed in Myl3 and Tnni1. qPCR was used to measure the expression of targeted molecules in gastrocnemius muscle. Results are analyzed and expressed as in Figure 3. ns signifies not significant, * *p* < 0.05, ** *p* < 0.01, *** *p* < 0.001.

In a previous study, we identified twenty different genes that play a role in energy metabolism, organismal injury and abnormalities, as well as the development and function of skeletal, muscular, and nervous systems by performing RNAseq analysis on gastrocnemius samples from *Ctns^−/−^* mice and WT mice [19]. For Myl3 and Tnni1, no significant changes were detected. Notable, repletion of 25(OH)D_3_ improved or normalized (Ankrd2, Csrp3, Cyfip2, Fhl1, Ly6a, Mup1, Myl2, Pdk4, Sell, Sln, Spp1, Tnnc1 and Tpm3) as well as (Atf3, Cidea, Fos, Sncg and Tbc1d1) muscle gene expression, but repletion of 1,25(OH)_2_D_3_ did not, in *Ctns^−/−^* mice (Figure 9). Potential functional significance of these specific 18 differentially expressed muscle genes has been previously discussed [19].

## 4. Discussion

In this paper, we report novel findings of the metabolic advantages of 25(OH)D_3_ over 1,25(OH)_2_D_3_ repletion in *Ctns^−/−^* mice, a genetic model of INC. Importantly, the 25(OH)D_3_ supplementation protocol normalized serum concentration of 25(OH)D_3_ and significantly increased but not normalize serum concentration of 1,25(OH)_2_D_3_ in *Ctns*^−/−^ mice whereas the 1,25(OH)_2_D_3_ supplementation protocol normalized serum concentration of 1,25(OH)_2_D_3_ but did not change the serum concentration of 25(OH)D_3_ in *Ctns*^−/−^ mice. At these administration dosages, 25(OH)D_3_ repletion corrected cachexia as well as attenuated fat and muscle pathologies in *Ctns^−/−^* mice, but 1,25(OH)_2_D_3_ repletion did not.

The metabolic advantages that accompanied 25(OH)D_3_ repletion over 1,25(OH)2D_3_ repletion in *Ctns^−/−^* mice involve many pathways. The main mechanism of 25(OH)D_3_ action likely results from local hydroxylation to 1,25(OH)_2_D_3_. Autocrine and paracrine effects may be involved as 1α-hydroxylase as well as VDR are present locally in target tissues such as skeletal muscle and fat. In addition, provision of more substrate such as 25(OH)D_3_ to the kidney will increase renal 1α-hydroxylation, accounting for the increase of circulating 1,25(OH)_2_D_3_. Thus, 25(OH)D_3_ supplementation has dual effects of increasing 1,25(OH)_2_D_3_ both locally and systemically. Furthermore, due to its hydrophobic nature, 25(OH)D_3_ potentially has increased cellular uptake compared to 1,25(OH)_2_D_3_. Cellular uptake of 25(OH)D_3_ occurs through the endocytosis of 25(OH)D_3_ to its binding complex mediated by megalin [34,35]. Furthermore, circulating 25(OH)D_3_ has much longer half-life (approximately two to three weeks) than 1,25(OH)_2_D_3_ (less than four hours) [20,36]. In several cell types, 25(OH)D_3_ at physiological concentrations has a similar level of potency compared with 1,25(OH)_2_D_3_ at pharmacological concentrations [22,23,24,25,26,27]. Even when it is not hydroxylated, 25(OH)D_3_ is an active hormone (as shown by the inhibition of 1-α hydroxylase) in various types of cells [22,25,26,27,37]. 25(OH)-19-nor-D_3_, a 25(OH)D_3_ analog exhibits anti-proliferative activity that is dependent on VDR but independent of 1α-hydroxylation [37]. Furthermore, 24-hydroxylase catalyzes the conversion of 25(OH)D_3_ and 1,25(OH)_2_D_3_ to 24R,25(OH)_2_D and 1,24,25-(OH)_3_D_3_, respectively [36,37]. Since distinct biological effects have been described for both 24R,25(OH)_2_D and 1,24,25-(OH)_3_D_3_ in numerous tissues and cell lines [37,38], the extent to which 25(OH)D_3_ acts directly or through its metabolites, such as 24R,25(OH)_2_D and 1,24,25-(OH)_3_D_3_, is unclear [39]. Therefore, a comprehensive system biology analysis is needed in future studies to further characterize the beneficial metabolic effects that resulted from 25(OH)D_3_ supplementation.

We showed the impact of 25(OH)D_3_ repletion in correcting cachexia and in vivo muscle function in *Ctns^−/−^* mice. These results may have translational importance. Anorexia and increased energy use at rest are associated with poor survival in subjects on chronic dialysis [40,41].

UCPs regulates energy metabolism for the entire body [42]. Upregulation of adipose and muscle UCPs has been described in cachexia from different diseases and thought to be mechanistic involved in hypermetabolism in these disorders [43,44]. UCPs, mitochondrial inner membrane proteins, produce heat while ATPases, proton channels located in the same membrane, generate ATP. Increased expression of UCPS not only stimulates the process of thermogenesis but also inhibits the synthesis of ATP [42]. Compared to the repletion of 1,25(OH)_2_D_3_, 25(OH)D_3_ repletion in *Ctns^−/−^* mice not only normalized fat UCP1 and muscle UCP3 levels but also significantly increased their ATP content. Murine fat and human cells all expressed VDR and 1α hydroxylase, the local enzyme that hydroxylates 25(OH)D_3_ to 1,25(OH)_2_D_3_ [45,46,47]. When mouse 3T3-L1 pre-adipocytes were incubated with 25(OH)D_3_, the media showed a buildup of 1,25(OH)_2_D_3_ [48]. 25(OH)D_3_ also binds to the UCP3 promoter region to modulate its expression in muscle [49]. WAT of *Ctns^−/−^* mice, there show upregulated thermogenic genes (Ppargc1α, Pgc1α, Cidea, Prdm16, and Dio2) (Figure 4), which was attenuated or normalized with 25(OH)D_3_ repletion.

Injury stimulates muscle satellite cells to differentiate and regenerate muscle fibers through activation of the transcription factor pair box 7 (Pax7) [50]. Compared to 1,25(OH)_2_D_3_ repletion, 25(OH)D_3_ repletion not only significantly decreased atrophy-related molecules but also significantly increased regenerative molecules in *Ctns^−/−^* mice (Figure 5).

Additionally, we documented morphological features in skeletal muscle of mice by measuring fiber diameter and fat deposition in gastrocnemius muscle. In *Ctns^−/−^* mice, 25(OH)D_3_ significantly improved muscle diameter and decreased fat deposition whereas 1,25(OH)_2_D_3_ did not (Figure 6 and Figure 7).

INC results from cystine accumulation primarily in kidney with many comorbidities [2,3]. Myopathy is prevalent in long term follow up studies in INC patients, including those who were treated with cysteamine. Gahl et al. [51] reported myopathy in 50% of 100 patients with INC; the incidence rising to 80% as the time of off-cysteamine therapy increased. Brodin-Sartoruius et al. reported myopathy in 22 out of 86 adult INC patients who were treated with cysteamine in a more recent long-term follow up study [52]. We measured muscle cystine content in our experimental animals. Muscle cystine content was significantly increased in *Ctns^−/−^* mice and repletion of 25(OH)D_3_ or 1,25(OH)_2_D_3_ did not change muscle cystine content in *Ctns^−/−^* mice (Figure 8). This would suggest that muscle wasting in INC is not the direct consequence of cystine accumulation.

Repletion of 25(OH) normalized or decreased muscle inflammatory cytokine expression in *Ctns^−/−^* mice (Figure 5). Inflammation may interact with oxidative stress, abnormal autophagy, apoptosis, defective endocystic trafficking, impaired proteolysis as well as mitochondrial dysfunction in cystinotic cells [53,54]. We will plan future research to address these potential pathways.

Finally, we used RNAseq analysis to assess the muscle transcriptome. Importantly, 25(OH)D_3_, but not 1,25(OH)_2_D_3_, significantly improved the abnormal signature of muscle genes (13 upregulated and 5 downregulated) in *Ctns^−/−^* mice (Figure 9). Ankrd2, Csrp3, Cyfip2, Fhl1, Ly6a, Spp1, and Tpm3 as well as Fos and Tbc1d1 are important determinants of muscle mass [19]. Mup1, Myl2, Pdk4, and Sln as well as Cidea and Sncg have been associated with energy metabolism.

## 5. Conclusions

**Figure 10:**
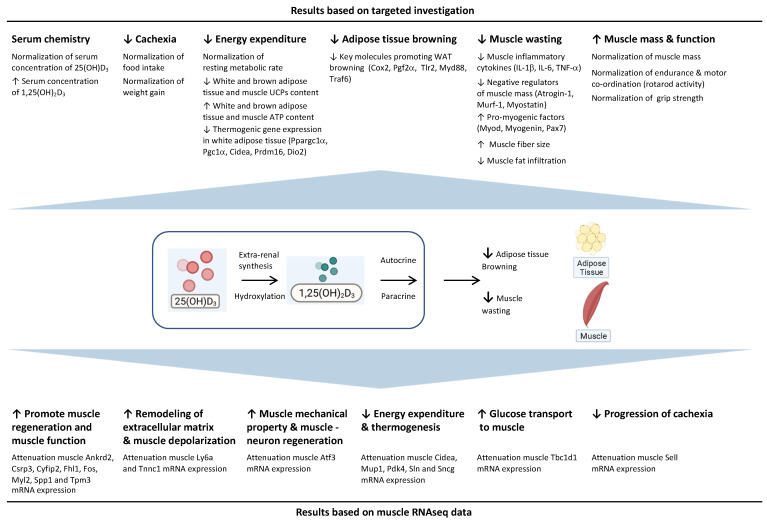
Summary of the metabolic advantages of repletion of 25(OH)D_3_ over repletion of 1,25(OH)_2_D_3_ in *Ctns^−/−^* mice. Created with BioRender.com, accessed on 7 September 2021.

Patients with INC exhibit diminished serum concentrations of 25(OH)D_3_ and 1,25(OH)_2_D_3_. In this study we demonstrated several metabolic advantages of 25(OH)D_3_ repletion over 1,25(OH)_2_D_3_ in *Ctns^−/−^* mice, a mouse model of INC, involving various cellular pathways (Figure 10). Monitoring and maintaining sufficient levels of circulating 25(OH)D_3_ and appropriate supplementation should be highlighted as a crucial treatment strategy in patients with INC to mitigate the devastating complications of adipose tissue browning and cachexia.

## Figures and Tables

**Table 1:** Serum and blood chemistry of mice. Twelve-month-old *Ctns^−/−^* mice and WT mice were treated with 25(OH)D_3_ (75 µg/kg/day), 1,25(OH)_2_D_3_ (60 ng/kg/day), or vehicle control (ethylene glycol) for six weeks. Data are expressed as mean ± SEM. Results of all five groups of mice were compared to those of WT + Vehicle mice, respectively. ^a^ *p* < 0.05, significantly different than WT + Vehicle mice. ^b^ *p* < 0.05, significantly different in *Ctns^−/−^* + 25(OH)D_3_ mice or *Ctns^−/−^* + 1,25(OH)_2_D_3_ mice relative to *Ctns^−/−^* + Vehicle mice.

	WT + Vehicle (*n* = 8)	WT + 25(OH)D_3_ (*n* = 8)	WT + 1,25(OH)_2_D_3_ (*n* = 8)	*Ctns^−/−^* + Vehicle (*n* = 8)	*Ctns^−/−^* + 25(OH)D_3_ (*n* = 8)	*Ctns^−/−^* + 1,25(OH)_2_D_3_ (*n* = 8)
BUN (mg/dL)	26.3 ± 4.3	27.9 ± 2.6	23.1 ± 4.3	67.6 ± 12.4 ^a^	57.6 ± 9.8 ^a^	65.7 ± 7.9 ^a^
Creatinine (mg/dL)	0.09 ± 0.02	0.08 ± 0.03	0.09 ± 0.02	0.24 ± 0.05 ^a^	0.23 ± 0.04 ^a^	0.21 ± 0.05 ^a^
Bicarbonate (mmol/L)	27.6 ± 2.3	27.8 ± 2.4	26.7 ± 2.7	26.7 ± 2.3	27.1 ± 5.6	26.7 ± 2.7
25(OH)D_3_ (ng/mL)	104.2 ± 13.5	105.3 ± 13.9	113.6 ± 12.8	43.6± 3.4 ^a^	109.4 ± 13.7 ^b^	58.9 ± 5.7 ^a^
1,25(OH)_2_D_3_ (pg/mL)	263.6 ± 31.5	201.7 ± 21.5	243.7 ± 12.8	125.6 ± 17.8 ^a^	193.4 ± 14.3 ^a,b^	276.1 ± 17.8 ^b^

**Table 2:** Serum and blood chemistry of mice. Twelve-month-old *Ctns^−/−^* mice and WT mice were treated with 25(OH)D_3_ (75 µg/kg/day), 1,25(OH)_2_D_3_ (60 ng/kg/day), or vehicle control (ethylene glycol) for six weeks. *Ctns^−/−^* + Vehicle mice were fed *ad libitum* whereas all other groups of mice were given the equivalent amount of energy intake as those of *Ctns^−/−^* + Vehicle mice. Results are expressed and analyzed as in Table 1. ^a^ *p* < 0.05, significantly different than WT + Vehicle mice. ^b^ *p* < 0.05, significantly different in *Ctns^−/−^* + 25(OH)D_3_ mice or *Ctns^−/−^* + 1,25(OH)_2_D_3_ mice relative to *Ctns^−/−^* + Vehicle mice.

	WT + Vehicle (*n* = 9)	WT + 25(OH)D_3_ (*n* = 9)	WT + 1,25(OH)_2_D_3_ (*n* = 9)	*Ctns^−/−^* + Vehicle (*n* = 9)	*Ctns^−/−^* + 25(OH)D_3_ (*n* = 9)	*Ctns^−/−^* + 1,25(OH)_2_D_3_ (*n* = 9)
BUN (mg/dL)	27.3 ± 4.3	22.7 ± 6.5	24.5 ± 2.5	65.9 ± 22.1 ^a^	75.4 ± 11.1 ^a^	76.9 ± 12.7 ^a^
Creatinine (mg/dL)	0.08 ± 0.04	0.09 ± 0.02	0.08 ± 0.03	0.21 ± 0.06 ^a^	0.26 ± 0.07 ^a^	0.28 ± 0.04 ^a^
Bicarbonate (mmol/L)	27.5 ± 2.6	27.1 ± 3.3	27.3 ± 2.4	26.7 ± 2.3	27.5 ± 4.3	26.9 ± 3.3
25(OH)D_3_ (ng/mL)	121.8 ± 23.5	124.1 ± 21.5	109.5 ± 17.6	48.2 ± 6.9 ^a^	125.4 ± 23.7 ^b^	64.5 ± 11.3 ^a^
1,25(OH)_2_D_3_ (pg/mL)	254.3 ± 24.3	213.6 ± 16.5	235.4 ± 23.6	126.4 ± 24.3 ^a^	189.8 ± 25.4 ^a,b^	254.3 ± 14.3 ^b^

## Data Availability

The authors confirm that the data supporting the findings of this study can be found in the article and Supplementary Materials. Additional raw data supporting the findings of this study can be made available by request from the corresponding author (R.H.M).

## Supplementary Materials

The following supporting information can be downloaded at: https://www.mdpi.com/article/10.3390/cells11203264/s1, Table S1: Immunoassay information for blood and serum chemistry, muscle adenosine triphosphate content as well as muscle and adipose tissue protein analysis. Table S2: PCR primer information. Tables S3–S5: Serum and blood chemistry of mice.

## References

[1] Town M.M., Jean G., Cherqui S., Attard M., Forestier L., Whitmore S.A., Callen D.F., Gribouval O., Broyer M., Bates G. (1998). A novel gene encoding an integral membrane protein is mutated in nephropathic cystinosis. Nat. Genet..

[2] Gahl W.A., Thoene J.G., Schneider J.A. (2002). Cystinosis. N. Engl. J. Med..

[3] Nesterova G., Gahl W. (2007). Nephropathic cystinosis: Late complications of a multisystemic disease. Pediatr. Nephrol..

[4] Theodoropoulos D.S., Krasnewich D., Kaiser-Kupfer M.I., Gahl W.A. (1993). Classic Nephropathic Cystinosis as an Adult Disease. JAMA.

[5] Cheung W.W., Cherqui S., Ding W., Esparza M., Zhou P., Shao J., Lieber R., Mak R.H. (2015). Muscle wasting and adipose tissue browning in infantile nephropathic cystinosis. J. Cachexia Sarcopenia Muscle.

[6] Fenzl A., Kiefer F.W. (2014). Brown adipose tissue and thermogenesis. Horm. Mol. Biol. Clin. Investig..

[7] Petruzzelli M., Schweiger M., Schreiber R., Campos-Olivas R., Tsoli M., Allen J., Swarbrick M., Rose-John S., Rincon M., Robertson G. (2014). A Switch from White to Brown Fat Increases Energy Expenditure in Cancer-Associated Cachexia. Cell Metab..

[8] A Vaitkus J., Celi F.S. (2016). The role of adipose tissue in cancer-associated cachexia. Exp. Biol. Med..

[9] Elattar S., Dimri M., Satyanarayana A. (2018). The tumor secretory factor ZAG promotes white adipose tissue browning and energy wasting. FASEB J..

[10] He Y., Liu R.-X., Zhu M.-T., Shen W.-B., Xie J., Zhang Z.-Y., Chen N., Shan C., Guo X.-Z., Lu Y.-D. (2019). The browning of white adipose tissue and body weight loss in primary hyperparathyroidism. EBioMedicine.

[11] Kong X., Yao T., Zhou P., Kazak L., Tenen D., Lyubetskaya A., Dawes B.A., Tsai L., Kahn B.B., Spiegelman B.M. (2018). Brown adipose tissue controls skeletal muscle function via the secretion of myostatin. Cell Metab..

[12] Kir S., White J.P., Kleiner S., Kazak L., Cohen P., Baracos V.E., Spiegelman B.M. (2014). Tumour-derived PTH-related protein triggers adipose tissue browning and cancer cachexia. Nature.

[13] Kir S., Komaba H., Garcian A.P., Economopoulos K.P., Liu W., Lanske B., Hodin R.A., Spiegelman B.M. (2015). PTH/PTHrP receptor mediates cachexia in models of kidney failure and cancer. Cell Metab..

[14] Autier P., Boniol M., Pizot C., Mullie P. (2014). Vitamin D status and ill health: A systematic review. Lancet Diabetes Endocrinol..

[15] Park J.E., Tirupathi Pichiah P.B., Cha Y.-S. (2018). Vitamin D and Metabolic Diseases: Growing Roles of Vitamin D. J. Obes. Metab. Syndr..

[16] Katzir Z., Shivil Y., Landau H., Kidrony G., Popovtzer M.M. (1988). Nephrogenic diabetes insipidus, cystinosis, and vitamin D. Arch. Dis. Child..

[17] Steinherz R., Chesney R.W., Schulman J.D., DeLuca H.F., Phelps M. (1983). Circulating vitamin D metabolites in nephropathic cystinosis. J. Pediatr..

[18] Chesney R.W., Hamstra J., Mazess R.B., Rose P., DeLuca H.F. (1982). Circulating vitamin D metabolites concentrations in childhood renal disease. Kidney Int..

[19] Cheung W.W., Hao S., Wang Z., Ding W., Zheng R.H., Gonzalez A., Zhan J.-Y., Zhou P., Li S.P., Esparza M.C. (2020). Vitamin D repletion ameliorates adipose tissue browning and muscle wasting in infantile nephropathic cystinosis-associated cachexia. J. Cachexia Sarcopenia Muscle.

[20] Querfeld U., Mak R.H. (2010). Vitamin D deficiency and toxicity in chronic kidney disease: In search of the therapeutic window. Pediatr. Nephrol..

[21] Dusso A.S. (2011). Kidney disease and vitamin D levels: 25-hydroxyvitamin D, 1,25-dihydroxyvitamin D, and VDR activation. Kidney Int. Suppl..

[22] Lou Y.-R., Laaksi I., Syvala A., Blauer M., Tammela T.L.J., YLikomi T., Tuohimaa P. (2004). 25-hydroxyvitamin D3 is an active hormone in human primary prostatic stromal cells. FASEB J..

[23] Peng X., Hawthorne M., Vaishnav A., St-Arnaud R., Mehta R.G. (2009). 25-Hydroxyvitamin D3 is a natural chemopreventive agent against carcinogen induced precancerous lesions in mouse mammary gland organ culture. Breast Cancer Res. Treat..

[24] Lou Y.-R., Molnar F., Perakyla M., Qiao S., Kalueff A.V., St-Arnaud R., Carlberg C., Tuohimaa P. (2010). 25-Hydroxyvitamin D(3) is an agonistic vitamin D receptor ligand. J. Steroid Biochem. Mol. Biol..

[25] Ritter C.S., Armbrecht H.J., Slatopolsky E., Brown A.J. (2006). 25-Hydroxyvitamin D(3) suppresses PTH synthesis and secretion by bovine parathyroid cells. Kidney Int..

[26] Zhang Z.L., Ding X.F., Tong J., Li B.Y. (2011). Partial rescue of the phenotype in 1alpha-hydroxylase gene knockout mice by vitamin D3 injection. Endocr. Res..

[27] Tuohimaa P., Wang J.-H., Khan S., Kuuslahti M., Qian K., Manninen T., Auvinen P., Vihinen M., Lou Y.-R. (2013). Gene expression profiles in human and mouse primary cells provide new insights into the differential actions of vitamin D3 metabolites. PLoS ONE.

[28] Young S., Struys E., Wood T. (2007). Quantification of creatine and guanidinoacetate using GC-MS and LC-MS/MS for the detection of cerebral creatine deficiency syndromes. Curr. Protoc. Hum. Genet..

[29] Du bowitz V., Sewry C.A., Oldfors A., Lane R.J.M. (2013). Muscle Biopsy: A Practical Approach.

[30] Mehlem A., Hagberg C., Muhl L., Eriksson U., Falkevall A. (2013). Imaging of neutral lipids by oil red O for analyzing the metabolic status in health and disease. Nat. Protoc..

[31] Cherqui S., Sevin C., Hamard G., Kaatzis V., Sich M., Pequignot M.O., Gogat K., Abitbol M., Broyer M., Gubler M.-C. (2002). Intralysosomal cystine Accumulation in Mice Lacking Cystinosin, the Protein Defective in Cystinosis. Mol. Cell Biol..

[32] Syres K., Harrison F., Tedlock M., Jester J.-V., Simpson J., Roy S., Salomon D.R., Cherqui S. (2009). Successful treatment of the murine model of cystinosis using bone marrow cell transplantation. Blood.

[33] Xie P. (2013). TRAF molecules in cell signaling and in human diseases. J. Mol. Signal..

[34] Hassan-Smith Z., Jenkinson C., Smith D.J., Hernadez I., Morgan S.A., Crabtree N.J., Gittoes N.J., Keevil B.G., Stewart P.M., Hewison M. (2017). 25-hydroxyvitamin D3 and 1,25-dihydroxyvitamin D3 exert distinct effects on human skeletal muscle function and gene expression. PLoS ONE.

[35] Atkins G.J., Anderson P.H., Findlay D.M., Welldon K.J., Vincent C., Zannettino A.C.W., O’Loughlin P.D., Morris H.A. (2007). Metabolism of vitamin D3 in human osteoblasts: Evidence for autocrine and paracrine activities of 1 alpha,25-dihydroxyvitamin D3. Bone.

[36] Boullata J.I. (2010). Vitamin D supplementation: A pharmacologic perspective. Curr. Opin. Clin. Nutr. Metab. Care.

[37] Askeno A., Saikatsu S., Kawane T., Horiuchi N. (1997). Mouse vitamin D-24-hydroxylase: Molecular cloning, tissue distribution, and transcriptional regulation by 1alpha,25-dihydroxyvitamin D3. Endocrinology.

[38] Munetsuna E., Kawanami R., Nishikawa M., Ikeda S., Nakabayashi S., Yasuda K., Ohta M., Kamakura M., Ikushiro S., Sakaki T. (2014). Anti-proliferative activity of 25-hydroxyvitamin D3 in human prostate cells. Mol. Cell. Endocrinol..

[39] Horst R.L., Wovkulich P.M., Baggiolini E.G., Uskoković. M.R., Engstrom G.W., Napoli J.L. (1984). (23S)-1,23,25-Trihydroxyvitamin D3: Its biologic activity and role in 1 alpha,25-dihydroxyvitamin D3 26,23-lactone biosynthesis. Biochemistry.

[40] Ikizler T.A., Wingard R.L., Sun M., Harvell J., Parker R.A., Hakim R.M. (1996). Increased energy expenditure in hemodialysis patients. J. Am. Soc. Nepohrol..

[41] Wang A.Y.-M., Sea M.M.-M., Tang N., Sanderson J.E., Lui S.-F., Li P.K.-T., Woo J. (2004). Resting energy expenditure and subsequent mortality risk in peritoneal dialysis patients. J. Am. Soc. Nephrol..

[42] Rousset S., Alves-Guerra M.-C., Mozo J., Miroux B., Cassard-Doulcier A.-M., Bouillaud F., Ricquier D. (2004). The biology of mitochondrial uncoupling proteins. Diabetes.

[43] Bing C., Brown M., King P., Collins P., Tisdsale M.J., Williams G. (2000). Increased gene expression of brown fat uncoupling protein (UCP)1 and skeletal muscle UCP2 and UCP3 in MAC16-induced cancer cachexia. Cancer Res..

[44] Wong K.E., Szeto F.L., Zhang W., Ye H., Kong J., Zhang Z., Sun X.J., Li Y.C. (2009). Involvement of the vitamin D receptor in energy metabolism: Regulation of uncoupling proteins. Am. J. Physiol. Endocrinol. Metab..

[45] Kamei Y., Kawada T., Kazuki R., Ono T., Kato S., Sugimoto E. (1993). Vitamin D receptor gene expression is up-regulated by 1, 25-dihydroxyvitamin D3 in 3T3-L1 preadipocytes. Biochem. Biophys. Res. Commun..

[46] Wang P.-Q., Pan D.-X., Hu C.-Q., Zhu Y.-L., Liu X.-J. (2020). Vitamin D–vitamin D receptor system down-regulates expression of uncoupling proteins in brown adipocyte through interaction with hairless protein. Biosci. Rep..

[47] Jonas M.I., Kurylowicz A., Bartoszewicz Z., Lisik W., Jonas M., Kozniewski K., Puzianowska-Kuznicka M. (2019). Vitamin D receptor gene expression in adipose tissue of obese individuals is regulated by miRNA and correlates with the pro-inflammatory cytokine level. Int. J. Mol. Sci..

[48] Nimitphong H., Holick M.F., Fried S.K., Lee M.-J. (2012). 25-hydroxyvitamin D3 and 1,25-dihydroxyvitamin D3 promote the differentiation of human subcutaneous preadipocytes. PLoS ONE.

[49] Fan Y., Futawaka K., Komaya R., Fukuda Y., Hayashi M., Imamoto M., Miyawaki T., Kasahara M., Tagami T., Moriyama K. (2016). Vitamin D3/VDR resists diet-induced obesity by modulating UCP3 expression in muscle. J. Biomed. Sci..

[50] Mierzejewski B., Archacka K., Grabowska I., Florkowska A., Ciemerych M.A., Brzoska E. (2020). Human and mouse skeletal muscle stem and progenitor cells in health and disease. Semin. Cell Devel. Biol..

[51] Gahl W.A., Balog J.Z., Kleta R. (2007). Nephropathic cystinosis in adults: Natural history and effects of oral cysteamine therapy. Ann. Intern. Med..

[52] Brodin-Sartorius A., Tete M.J., Niaudet P., Antignac C., Guest G., Ottolenghi C., Charbit M., Moyse D., Legendre C., Lesavre P. (2012). Cysteamine therapy delays the progression of nephropathic cystinosis in late adolescents and adults. Kidney Int..

[53] Ruivo R., Anne C., Sagne C., Gasnier B. (2009). Molecular and cellular basis of lysosomal transmembrane protein dysfunction. Biochim. Biophys. Acta.

[54] Elmonem M.A., Veys K.R.P., Prencipe G. (2022). Nephropathic cystinosis: Pathogenic roles of inflammation and potential for new therapies. Cells.

