# Supplementary material for: Metabolic Advantage of 25(OH)D3 versus 1,25(OH)2D3 Supplementation in Infantile Nephropathic Cystinosis-Associated Adipose Tissue Browning and Muscle Wasting
Source: Cells. 2022 Oct 17;11(20):3264. doi: 10.3390/cells11203264 (PMC9600749; doi:10.3390/cells11203264)
Supplement: Supplementary file 1 [file cells-11-03264-s001.zip › cells-1751509-supplementary.pdf]

Table S1. Immunoassay information for blood and serum chemistry, muscle adenosine triphosphate content as well as muscle and adipose tissue protein analysis.

Blood & Serum chemistry	Assay information
Bicarbonate & BUN	VetScan Comprehensive Diagnostic Profile, Abaxix, 500-0038
Creatinine	LC-MS/MS method
25(OH)D_3	IDS, AC-35F1
1,25$(OH)_2D_3$	IDS, AC-62F1

Muscle & adipose tissue	Assay information
ATP Assay Kit	Abcam, ab83355
Mouse UCP1 ELISA kits	Aviva Systems Biology, OKCD02970
Mouse UCP3 ELISA kits	Aviva Systems Biology, OKEH05259

Table S2. PCR primer information.

Gene	Forward primer	Reverse primer
Ankrd2	TGGACATGCTAGTGCTAGAGG	CGCTTTTTCTGCTTGCGTTTT
Atf3	GAGGATTTTGCTAACCTGACACC	TTGACGGTAACTGACTCCAGC
Atrogin-1	CAGCTTCGTGAGCGACCTC	GGCAGTCGAGAAGTCCAGTC
Cidea	TGACATTCATGGGATTGCAGAC	GGCCAGTTGTGATGACTAAGAC
CD137	CGTGCAGAACTCCTGTGATAAC	GTCCACCTATGCTGGAGAAGG
Cox2	AACCCAGGGGATCGAGTGT	CGCAGCTCAGTGTTTGGGAT
Csrp3	GGGGGAGGTGCAAAATGTG	CAGGCCATGCAGTGGAAACA
Cyfip2	ATGACCACCCACGTCACTTTG	CCTGTCCTCGAAGTTCGTGTC
Dio2	AATTATGCCTCGGAGAAGACCG	GGCAGTTGCCTAGTGAAAGGT
Fhl1	GACTGCCGCAAGCCCATAA	CCAAGGGGTGAAGGCACTT
Fos	TTGAGCGATCATCCCGGTC	GCGTGAGTCCATACTGGCAAG
IL1β	GCAACTGTTCCTGAACTCAACT	ATCTTTTGGGGTCCGTCAACT
IL6	TAGTCCTTCCTACCCCAATTTCC	TTGGTCCTTAGCCACTCCTTC
Ly6a	AGGAGGCAGCAGTTATTGTGG	CGTTGACCTTAGTACCCAGGA
Mup1	GAAGCTAGTTCTACGGGAAGGA	AGGCCAGGATAATAGTATGCCA
Murf-1	GTGTGAGGTGCCTACTTGCTC	GCTCAGTCTTCTGTCCTTGGA
Myd88	TCATGTTCTCCATACCCTTGGT	AAACTGCGAGTGGGGTCAG
Myl2	ATCGACAAGAATGACCTAAGGGA	ATTTTTCACGTTCACTCGTCCT
Myl3	TGGGGAAGCCAAAACAGGAAG	AGCCATCAGTTTCTCTACCTCA
Myod	CCACTCCGGGACATAGACTTG	AAAAGCGCAGGTCTGGTGAG
Myogenin	GAGACATCCCCCTATTTCTACCA	GCTCAGTCCGCTCATAGCC
Myostatin	AGTGGATCTAAATGAGGGCAGT	GTTTCCAGGCGCAGCTTAC
Pax7	TCTCCAAGATTCTGTGCCGAT	CGGGGTTCTCTCTCTTATACTCC
Pdk4	AGGGAGGTCGAGCTGTTCTC	GGAGTGTTCACTAAGCGGTCA
Pgc1α	TATGGAGTGACATAGAGTGTGCT	GTCGCTACACCACTTCAATCC
Pgf2α synthase	CTGGACTCATCGCAAACACAA	AGGAAGCCTTTGACTTCTGTCTA
Ppargc1α	AGAGCCCCATCTGTCCTCTC	ACTGGTAGTCTGCAAAACCAAA
Prdm16	CCCCACATTCCGCTGTGAT	CTCGCAATCCTTGCACTCA
Sell	TACATTGCCCAAAAGCCCTTAT	CATCGTTCCATTTCCCAGAGTC
Sln	CTTGGGTGGATACCACGTCTG	CTTCTGTCTTACAGCCATAGCC
Sncg	AAAGACCAAGCAGGGAGTAACG	GACCACGATGTTTTCAGCCTC
Spp1	AGGAAGAAGTAGGCATTTCTGGT	TCGGCTCTGCAATGTTGTCTG
Tbx1	CTGTGGGACGAGTTCAATCAG	TTGTCATCTACGGGCACAAAG
Tbc1d1	TTCCTGGGGGTGAGTCTCAG	GCAGGGCATTACGGTAGGAG
Tlr2	GCAAACGCTGTTCTGCTCAG	AGGCGTCTCCCTCTATTGTATT
Tmem26	TTCCTGTTGCATTCCCTGGTC	GCCGGAGAAAGCCATTTGT
TNF-α	CCCTCACACTCAGATCATCTTCT	GCTACGACGTGGGCTACAG
Tnnc1	GCGGTAGAACAGTTGACAGAG	CCAGCTCCTTGGTGCTGAT
Tnni1	ATGCCGGAAGTTGAGAGGAAA	TCCGAGAGGTAACGCACCTT
Tpm3	ACCACCATCGAGGCGGTAA	CCCTTTCCTCCGCATCATCA
Traf6	AAAGCGAGAGATTCTTTCCCTG	ACTGGGGACAATTCACTAGAGC
Gapdh (internal control)	AGGTCGGTGTGAACGGATTTG	TGTAGACCATGTAGTTGAGGTCA

Table S3. Serum and blood chemistry of mice. Twelve-month-old *Ctns*$^{-/-}$ mice and WT mice were treated with 25(OH)D_3 (25 µg/kg/day), 1,25(OH)$_2$$D_3$ (20 ng/kg/day) or vehicle control (ethylene glycol) for six weeks. All mice were fed *ad libitum*. Data are expressed as mean ± SEM. Results of *Ctns*$^{-/-}$ + Vehicle, *Ctns*$^{-/-}$ + 25(OH)D_3 and *Ctns*$^{-/-}$ + 1,25(OH)$_2$$D_3$ mice were compared to those of WT + Vehicle mice, respectively. [a] $p < 0.05$, significantly different in *Ctns*$^{-/-}$ mice than WT mice. [b] $p < 0.05$, significantly different in *Ctns*$^{-/-}$ + 25(OH)D_3 or *Ctns*$^{-/-}$ + 1,25(OH)$_2$$D_3$ mice versus *Ctns*$^{-/-}$ + Vehicle mice. BUN, blood urea nitrogen.

	WT + Vehicle (n = 6)	*Ctns*$^{-/-}$ + Vehicle (n = 5)	*Ctns*$^{-/-}$ + 25(OH)D_3 (n = 6)	*Ctns*$^{-/-}$ + 1,25(OH)$_2$$D_3$ (n = 6)
BUN (mg/dL)	25.3 ± 3.8	74.3 ± 15.8 [a]	78.4 ± 21.4 [a]	69.5 ± 11.4 [a]
Creatinine (mg/dL)	0.11 ± 0.03	0.26 ± 0.04 [a]	0.24 ± 0.06 [a]	0.27 ± 0.09 [a]
Bicarbonate (mmol/L)	26.5 ± 2.7	26.8 ± 3.2	26.8 ± 2.3	27.1 ± 2.2
25(OH)D_3 (ng/mL)	103.5 ± 22.5	38.5 ± 6.3 [a]	55.6 ± 6.8 [a,b]	43.2 ± 5.3 [a]
1,25(OH)$_2$$D_3$ (pg/mL)	255.6 ± 35.4	98.6 ± 25.4 [a]	113.1 ± 14.6 [a]	158.4 ± 11.3 [a,b]

Table S4. Serum and blood chemistry of mice. Twelve-month-old *Ctns*$^{-/-}$ mice and WT mice were treated with 25(OH)D_3 (50 μg/kg/day), 1,25(OH)$_2D_3$ (40 ng/kg/day) or vehicle control (ethylene glycol) for six weeks. All mice were fed *ad libitum*. Results are expressed and analyzed as in Supplemental Table S3. ^{a} $p < 0.05$, significantly different in *Ctns*$^{-/-}$ mice than WT mice. ^{b} $p < 0.05$, significantly different in *Ctns*$^{-/-}$ + 25(OH)D_3 or *Ctns*$^{-/-}$ + 1,25(OH)$_2D_3$ mice versus *Ctns*$^{-/-}$ + Vehicle mice.

	WT + Vehicle (n = 6)	*Ctns*$^{-/-}$ + Vehicle (n = 5)	*Ctns*$^{-/-}$ + 25(OH)D_3 (n = 6)	*Ctns*$^{-/-}$ + 1,25(OH)$_2D_3$ (n = 6)
BUN (mg/dL)	25.3 ± 3.8	74.3 ± 15.8 ^{a}	78.4 ± 21.4 ^{a}	69.5 ± 11.4 ^{a}
Creatinine (mg/dL)	0.11 ± 0.03	0.26 ± 0.04 ^{a}	0.24 ± 0.06 ^{a}	0.27 ± 0.09 ^{a}
Bicarbonate (mmol/L)	26.5 ± 2.7	26.8 ± 3.2	26.8 ± 2.3	27.1 ± 2.2
25(OH)D_3 (ng/mL)	103.5 ± 22.5	38.5 ± 6.3 ^{a}	55.6 ± 6.8 ^{a,b}	43.2 ± 5.3 ^{a}
1,25(OH)$_2D_3$ (pg/mL)	255.6 ± 35.4	98.6 ± 25.4 ^{a}	113.1 ± 14.6 ^{a}	158.4 ± 11.3 ^{a,b}

Table S5. Serum and blood chemistry of mice. Twelve-month-old *Ctns*$^{-/-}$ mice and WT mice were treated with 25(OH)D_3 (75 µg/kg/day), 1,25$(OH)_2D_3$ (60 ng/kg/day), or vehicle control (ethylene glycol) for six weeks. All mice were fed *ad libitum*. Results are expressed and analyzed as in Supplemental Table S3. ^{a} $p < 0.05$, significantly different in *Ctns*$^{-/-}$ mice than WT mice. ^{b} $p < 0.05$, significantly different in *Ctns*$^{-/-}$ + 25(OH)D_3 or *Ctns*$^{-/-}$ + 1,25$(OH)_2D_3$ mice versus *Ctns*$^{-/-}$ + Vehicle mice.

	WT<br>+ Vehicle<br>(*n* = 4)	*Ctns*$^{-/-}$<br>+ Vehicle<br>(*n* = 4)	*Ctns*$^{-/-}$<br>+ 25(OH)D_3<br>(*n* = 6)	*Ctns*$^{-/-}$<br>+ 1,25$(OH)_2D_3$<br>(*n* = 6)
BUN (mg/dL)	23.5 ± 6.9	57.8 ± 5.3 ^{a}	67.8 ± 4.8 ^{a}	74.3 ± 11.1 ^{a}
Creatinine (mg/dL)	0.11 ± 0.03	0.26 ± 0.04 ^{a}	0.28 ± 0.06 ^{a}	0.29 ± 0.07 ^{a}
Bicarbonate (mmol/L)	27.4 ± 2.1	27.5 ± 1.6	27.3 ± 14	26.5 ± 2.2
25(OH)D_3 (ng/mL)	109.6 ± 17.8	38.7 ± 5.9 ^{a}	114.3 ± 14.3 ^{b}	53.4 ± 11.5 ^{a}
1,25$(OH)_2D_3$ (pg/mL)	274.3 ± 17.5	115.3 ± 21.4 ^{a}	201.7 ± 21.5 ^{a}	265.1 ± 11.8 ^{b}
